# Perspectives on ankle-foot technology for improving gait performance of children with Cerebral Palsy in daily-life: requirements, needs and wishes

**DOI:** 10.1186/s12984-023-01162-3

**Published:** 2023-04-12

**Authors:** Cristina Bayón, Marleen van Hoorn, Antonio Barrientos, Eduardo Rocon, Joyce P. Trost, Edwin H. F. van Asseldonk

**Affiliations:** 1 Department of Biomechanical Engineering, University of Twente, Enschede, The Netherlands; 2 Centro de Automática y Robótica, Universidad Politécnica de Madrid, Consejo Superior de Investigaciones Científicas, Madrid, Spain; 3 Gillette Children’s, Saint Paul, MN USA

**Keywords:** Ankle-foot orthosis, Cerebral Palsy, Gait, Daily-life, Assistive technology

## Abstract

**Background:**

Ankle-foot orthoses (AFOs) are extensively used as a primary management method to assist ambulation of children with Cerebral Palsy (CP). However, there are certain barriers that hinder their prescription as well as their use as a mobility device in all kinds of daily-life activities. This exploratory research attempts to further understand the existing limitations of current AFOs to promote a better personalization of new design solutions.

**Methods:**

Stakeholders’ (professionals in CP and end-users with CP) perspectives on AFO technology were collected by two online surveys. Respondents evaluated the limitations of current assistive solutions and assessment methods, provided their expectations for a new AFO design, and analyzed the importance of different design features and metrics to enrich the gait performance of these patients in daily-life. Quantitative responses were rated and compared with respect to their perceived importance. Qualitative responses were classified into themes by using content analysis.

**Results:**

130 survey responses from ten countries were analyzed, 94 from professionals and 36 from end-users with CP. The most highly rated design features by both stakeholder groups were the comfort and the ease of putting on and taking off the assistive device. In general, professionals preferred new features to enrich the independence of the patient by improving gait at functional levels. End-users also considered their social acceptance and participation. Health care professionals reported a lack of confidence concerning decision-making about AFO prescription. To some degree, this may be due to the reported inconsistent understanding of the type of assistance required for each pathological gait. Thus, they indicated that more information about patients’ day-to-day walking performance would be beneficial to assess patients’ capabilities.

**Conclusion:**

This study emphasizes the importance of developing new approaches to assess and treat CP gait in daily-life situations. The stakeholders’ needs and criteria reported here may serve as insights for the design of future assistive devices and for the follow-up monitoring of these patients.

**Supplementary Information:**

The online version contains supplementary material available at 10.1186/s12984-023-01162-3.

## Background

Physical disabilities derived from neurological or motor disorders are a global societal problem. In children, Cerebral Palsy (CP) is the major cause of physical disability such as gait limitations [1]. CP results from damage to the child’s brain during birth or early childhood, which may lead to permanent neurological impairments related to motor control, strength, muscle dysfunction, balance and/or posture [2]. According to the Cerebral Palsy Alliance Research Foundation [3], 18 million people are living with CP worldwide, with an estimated lifetime care cost of around €1 million per individual [4]. This implies a real impact on the individual, and a true financial burden for the families in particular and society in general [5, 6].

The improvement of walking ability is considered one of the primary goals to allow for a more active and independent lifestyle in CP [2, 7]. In conjunction with other medical, surgical, and therapeutic interventions, assistive devices are essential in the management of gait and mobility of these patients [7–9].

Due to the role of the ankle joint in gait [7] and the greater muscle dysfunction of distal lower-extremity muscles in CP [10, 11], ankle-foot orthoses (AFOs) are the foremost used type of assistive devices [7–9, 12–15]. Technological advances over the last decades have resulted in the development of AFO designs for CP, which are typically prescribed depending on (1) the pathological gait pattern and (2) the functional capacities (level of the Gross Motor Function Classification System, GMFCS) of the child [8, 9]. As such, based on the literature [2, 8, 15–17] it is possible to obtain a general relationship between the level of motor impairments and the most commonly recommended AFO solutions and other management methods (Appendix A, Table 4). However, this general relationship is too broad with respect to the *ideal* orthotic management that should be prescribed to tailor the specific needs of an individual patient. First, current clinical standards for choosing all possible AFO design features and the impact of these features on patient outcomes in daily-life are unclear [13, 18–21]. Second, the assessment of the patient’s walking *capacity* in a laboratory is not always representative of the patient’s walking *performance* in real life [20, 22]. Additional information about the patient’s community walking activities would be beneficial to better understand their gait problems and improve the design and prescription of the different AFO solutions [21].

Another crucial factor is that traditional AFO designs are normally passive and present considerable limitations related to decreasing push-off power, which is associated with an increased walking energy cost and compensations around the hip [12]. Recently, adjustable dynamic response AFOs (ADR-AFOs) have been introduced to the market, which provide greater adjustability of the AFO by the clinician to the specific patient’s needs. This type of instrumented orthosis allows variable ankle range of motion (ROM) and selective support for the tibialis anterior and gastrocnemius-soleus muscles, storing and returning energy during gait. Thus, they aim to make walking more natural and comfortable without increasing the patient’s energy cost of walking that is often associated to a limited push-off. Although ADR-AFOs introduce promising advances, the benefits are still variable depending on the type of patient and/or walking scenario [12–14, 23, 24]. One of the bigger limitations of ADR-AFOs is the difficulty of choosing the correct spring module (desired stiffness) for each patient [12]. Also, similar to traditional designs, ADR-AFOs present limited modularity (i.e. incapability to ‘grow up’ with the child) [25]. This, together with the poor adaptability to challenging mobility tasks and ground variations encountered during daily-life [7, 26], make them (still) ineffective solutions to be employed in all varieties of everyday activities [12, 24].

An emerging trend to address the shortcomings of previous AFO designs is the use of untethered robot-assisted AFOs [27, 28]. The possibilities of control and actuation of these solutions allow a wide range of adaptability to both the patient and the environment. Orekhov et al. recently presented some initial evidence supporting the effectiveness of using a robot-assisted AFO across different terrains for children and adults with CP [28]. However, drawbacks of using these device in daily-life include weight, bulkiness, comfort, battery duration and operability [27, 28]. For example, Yeung et al. reported an undesired effect of the current shortcomings: long-term use of a powered AFO (0.5 kg) resulted in a reduction of knee flexion during swing in patients with hemiparesis [27]. These effects conflict with the main purpose of the assisted AFO, which restricts their potential extension to continuous use in daily-life.

The purpose of this exploratory study is to better understand the current limitations of AFO technology for CP, aiming to identify areas of improvement for a better personalization of new design solutions. When the goal is to improve physical functions in CP, it is recommended [29] to first set the user-chosen goals and to focus on practice within a real-life context. According to these recommendations, here we assess and compare perspectives of stakeholders (professionals in CP and end-users with CP) on assistive technology for improving gait performance, with respect to perceived importance of design features, expectations for a new design and potential changes in current devices. This allows us to answer two main research questions: (1) What are considered the prioritized areas of improvement for ankle-foot orthosis to facilitate and enrich patients’ gait performance in daily-life activities?; and (2) Which real-world gait measures do clinicians find important to inform clinical decision-making when assessing patients’ progression and prescribing new assistive devices?

## Methods

### Study design

**Fig. 1:**
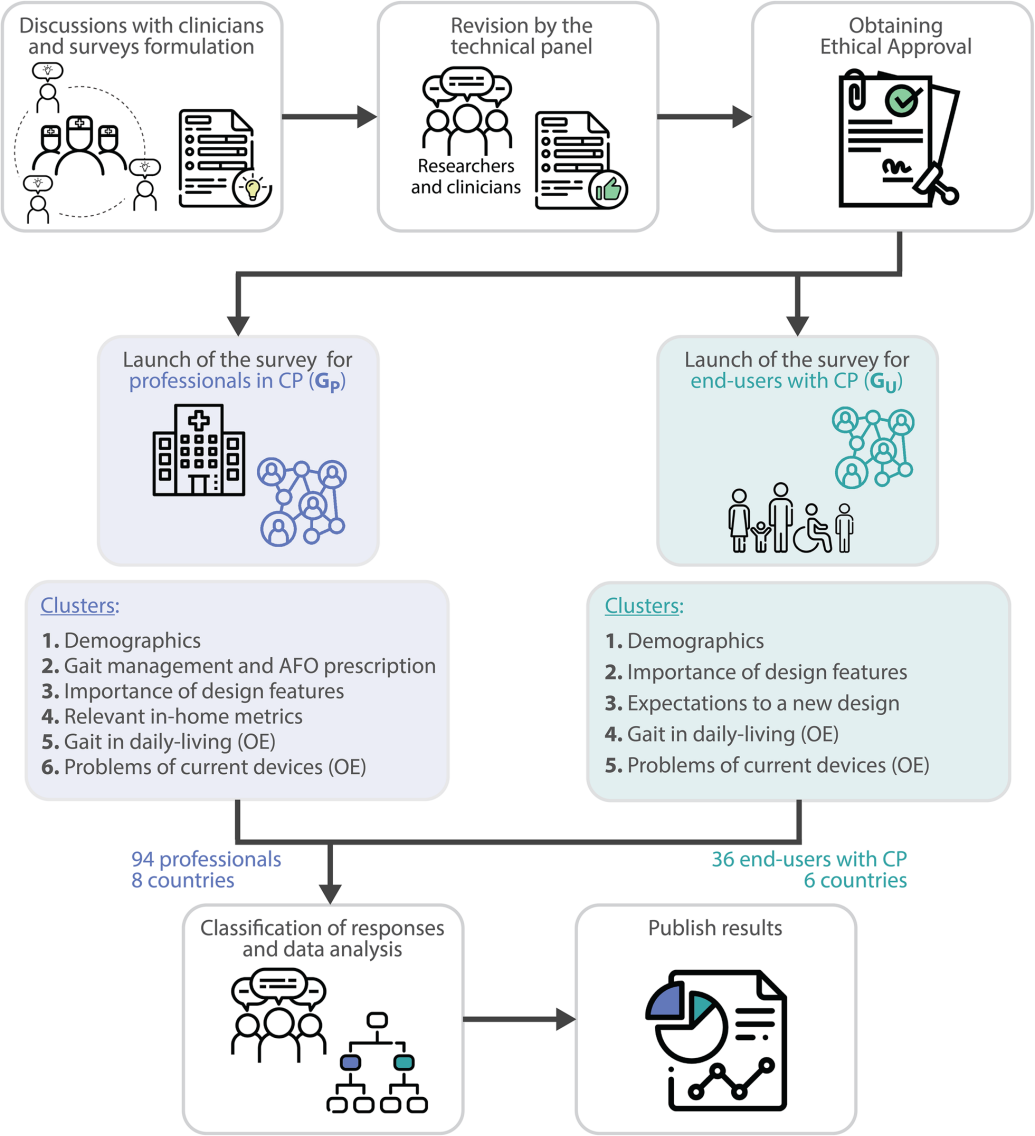
Surveys process. The affiliations comprising the technical panel were: University of Twente (UT), Spanish National Research Council (CSIC), Gillette Children’s (GC). OE stands for open-ended questions

An exploratory phenomenological mixed study was developed based on two online surveys (formulated in English following the flow-chart of Fig. 1). The surveys were intended to collect quantitative and qualitative answers from two stakeholder groups: (1) professionals who specialize in CP (G$_P$); and (2) end-users with CP (G$_U$). The final content of the surveys was designed based on the input provided through discussions with clinicians from different institutions^1^, previous literature [18, 30], and the experience of a technical panel comprising researchers and clinicians from the affiliations involved (University of Twente, Spanish National Research Council, Gillette Children’s).

Before launch, the surveys were piloted with two professionals and two end-users, who reviewed the corresponding questions and provided feedback for minor adaptations on wording and layout (e.g. several questions were accompanied by pictures to enhance the reader’s comprehension). Additionally, the authors CB and MvH had informal conversations with several professionals in the field and end-users with the main aim of clarifying possible unclear terms and expressions.

The research ethics board of the University of Twente approved the final English versions of the surveys (reference number 2021.91). These final versions can be found in the Additional files 1 and 2. The English versions were subsequently translated into Spanish and Dutch by native speakers (CB, MvH and EHFvA), who discussed the content and intent of the questions to facilitate the accurate translations.

All collected responses were anonymous, as no personal data was required. Participants gave consent for voluntary participation on filling in the questionnaires. The protocols for data protection of the affiliations involved were applied.

#### Survey for G$_P$: professionals in CP

The final version of the survey for group G$_P$ consisted of 57 questions classified into six clusters (see Additional file 1): (1) *Demographics*; (2) *Gait management and AFO prescription*, composed of questions that were only answered by those professionals working in the health care sector; (3) *Importance of design features*, with questions related to usability and aesthetic considerations, functional considerations, and practical considerations. Within this cluster, health care professionals were asked two extra questions related to which kind of assistance (i.e. support push-off, inhibit foot-slap and prevent drop-foot) would they apply to the different types of pathological gait in CP and to the five levels of the GMFCS; (4) *Relevant in-home metrics*, which included questions referring to general, temporal, spatial and ground clearance parameters; (5) *Gait in daily-living*, with an open-ended (OE) question “Which daily-life activities would benefit from improved gait performance in children with CP?”; and (6) *Limitations of current devices*, including two OE questions “What changes to the current exoskeletons are needed to improve walking in daily-life situations?” and “What changes to the current AFOs are needed to improve walking in daily-life situations?”.

Closed-ended (CE) questions primarily used a multiple-choice response format (*Demographics*), or a 5—point Likert scale, with Likert scales ranging from 1—strongly disagree to 5—strongly agree (*Gait management and AFO prescription*), or 1—very unimportant to 5—very important (*Importance of design features* and *Relevant in-home metrics*). In the latter case, after respondents selected the importance for each of the different factors, they were asked about their top-3 most important factors to differentiate in case they ranked all of them as ‘important’ or ‘very important’.

#### Survey for G$_U$: end-users with CP

The final version of the survey for group G$_U$ was composed of a total of 41 questions (Additional file 2), which were classified into five clusters: (1) *Demographics*; (2) *Importance of design features*, similarly to what was presented for G$_P$; (3) *Expectations for a new design*, whose questions were based and adapted from the Unified Theory of Acceptance and Use of Technology questionnaire (UTAUT)[29]. These referred to effort expectancy (EE, degree of ease associated with the use of the new system), performance expectancy (PE, degree to which the patient believes that using the new system will help them to attain gains in walking performance), social influence (SI, degree to which the patient’s behaviour is influenced by the way in which they believe others will view them as a result of using the new technology), and facilitating conditions (FC, degree to which the patient believes that daily-life infrastructures facilitate the use of the system); (4) *Gait in daily-living*; and (5) *Limitations of current devices*. Both clusters (4) and (5) included OE questions similar to those presented for G$_P$.

For G$_U$, we used a multiple-choice response format for the *Demographics*, and 5–point Likert scales, with 1—very unimportant to 5—very important for *Importance of design features*, and with 1—strongly disagree to 5—strongly agree for *Expectations for a new device*. Like G$_P$, respondents were asked about their top-3 most important factors in the cluster *Importance of design features* to differentiate in case they rated all of them as ‘important’ or ‘very important’.

### Participants and data collection

Two groups of stakeholders were approached to participate as respondents to the surveys: G$_P$ and G$_U$. Participants were recruited using snowball sampling. Data were collected between July and October, 2021.

The group of G$_P$ included professionals over 18 years of age who specialize in CP, with a special focus on the health care sector (i.e. physiotherapists, rehabilitation physicians, surgeons, orthotists), but also including others, such as non-clinical researchers or equipment vendors. The group of G$_U$ included end-users with CP of any age and functional skills (GMFCS levels I to V). In cases where patients were unable to answer the survey (e.g. too young to understand the questions, severe cognitive impairment), parents or legal caregivers gave their responses instead.

The study information for G$_P$, including the survey link, was primarily sent by email to eligible contacts within our international network. This comprised several hospitals and rehabilitation centers in Spain, The Netherlands and The United States, companies in The Netherlands and Germany, and other researchers affiliated with institutions in Spain, The Netherlands, The United States, Switzerland and Colombia. The invitations included a request to forward the link to other eligible professionals specialized in CP to maximize its distribution.

The main strategy to approach end-users (G$_U$) to complete the survey was through the previously contacted hospitals and rehabilitation centers.

Besides the distribution by email, we also advertised both surveys with flyers in hospital waiting rooms and on social media (i.e. Twitter).

### Data analysis

Responses to both surveys were exported into an Excel file for data cleaning and analysis. Spanish and Dutch responses were translated into English by two bilingual researchers (CB and MvH).

#### Closed-ended responses

Descriptive statistics and graphic representations were used to summarize and compare CE responses. For the cluster *Importance of design features*, the Mann–Whitney U test with $\alpha =0.05$ was performed in Matlab 2018b (Mathworks, Natick, MA, USA) to determine significant differences between both stakeholder groups.

#### Open-ended responses

Responses to the OE questions were analyzed using content analysis [31]. Irrelevant answers (e.g. “I don’t know”) were removed prior to starting the analysis. Data were imported into ATLAS.ti 9 (ATLAS.ti GmbH, Berlin, Germany), and responses were reread multiple times (by CB and MvH) to identify the key thoughts, impressions and concepts. The authors CB and MvH discussed emergent broad themes and subcategories, which were used to sub-categorize the responses using inductive coding. Frequencies of themes and subcategories were assessed. Responses could be coded with more than one theme.

## Results

### Participants

A total of 94 professionals and 36 end-users responded to the surveys. Demographic information about the respondents is described in Tables 1 and 2 for G$_P$ and G$_U$ respectively.

**Table 1:** Demographics of G$_P$ stakeholder

Professionals in CP	Frequency	Percent
(n$$_{G_P}$$ = 94)		
Sex		
Male	35	37.2
Female	59	62.8
Age		
18–24	0	0
25–34	22	23.4
35–44	37	39.4
45–54	22	23.4
55–64	9	9.6
65 or above	4	4.3
Country		
Spain	45	47.9
The Netherlands	31	33.0
USA	9	9.6
Belgium	2	2.1
Colombia	1	1.1
Ecuador	1	1.1
Mexico	1	1.1
Switzerland	1	1.1
Prefer not to answer	3	3.2
Profession		
Physiotherapist	50	53.2
Rehabilitation physician	17	18.1
Researcher	13	13.8
Surgeon	3	3.2
Equipment vendor	3	3.2
Orthotist	2	2.1
Other*	6	6.4
Time working in the field		
Less than 1 year	0	0
1–4 years	8	8.5
5–10 years	21	22.3
11+ years	65	69.1
Experience with AFOs for CP		
Yes	78	83.0
No	16	17.0
Experience with exo or ADR-AFO		
Yes	27	28.7
No	67	71.3

*Other professions such as occupational therapist,podologist

**Table 2:** Demographics of G$_U$ stakeholder

End-Users with CP	Frequency	Percent
(n$$_{G_U}$$ = 36)		
Sex		
Male	19	52.8
Female	16	44.4
Prefer not to disclose	1	2.8
Age		
Under 3	3	8.3
3–7	13	36.1
8–12	11	30.6
13–17	5	13.9
18 or above	3	8.3
Prefer not to answer	1	2.8
Country		
Spain	20	55.6
The Netherlands	5	13.9
USA	3	8.3
Belgium	3	8.3
Peru	2	5.6
Chile	1	2.8
Prefer not to answer	2	5.6
Level of GMFCS		
GMFCS I	7	19.4
GMFCS II	13	36.1
GMFCS III	3	8.3
GMFCS IV	9	25.0
GMFCS V	4	11.1
Experience with exo or ADR-AFO		
Yes	11	30.6
No	25	69.4
Type of AFO currently using*		
GRAFO	1	2.8
SAFO	15	41.7
HAFO	12	33.3
PLS-AFO	4	11.1
ADR-AFO	2	5.6
SPM	2	5.6

*See Appendix A for the description of AFO types

### Gait management and AFO prescription

Health care professionals (83 out of 94 total G$_P$ respondents) were asked to agree or disagree (1—strongly disagree to 5—strongly agree) with four statements about current gait management in CP and the challenges to assess specific user’s needs for their daily-life activities.

Only 44.6% of the health care professionals agreed (or strongly agreed) with the statement “There is enough information to feel confident when prescribing the correct AFO type (solid, hinged, ADR...) for a specific patient”, which indicates that more than 50% of the health care professionals think that more information is required.

As many as 79.3% of the health care respondents believed (i.e. agreed or strongly agreed) that “patients’ performance in the clinic is different than in real-life settings”. This might be related to the fact that almost all the health care professionals (98.8%) considered that “it would be important to get information about patients’ walking on daily-life activities”, and that 95.2% of them agreed with the statement “A report on the use of AFOs on daily-life could provide useful information to improve the patient’s assessment in clinic”.

### Rated importance of design features

**Fig. 2:**
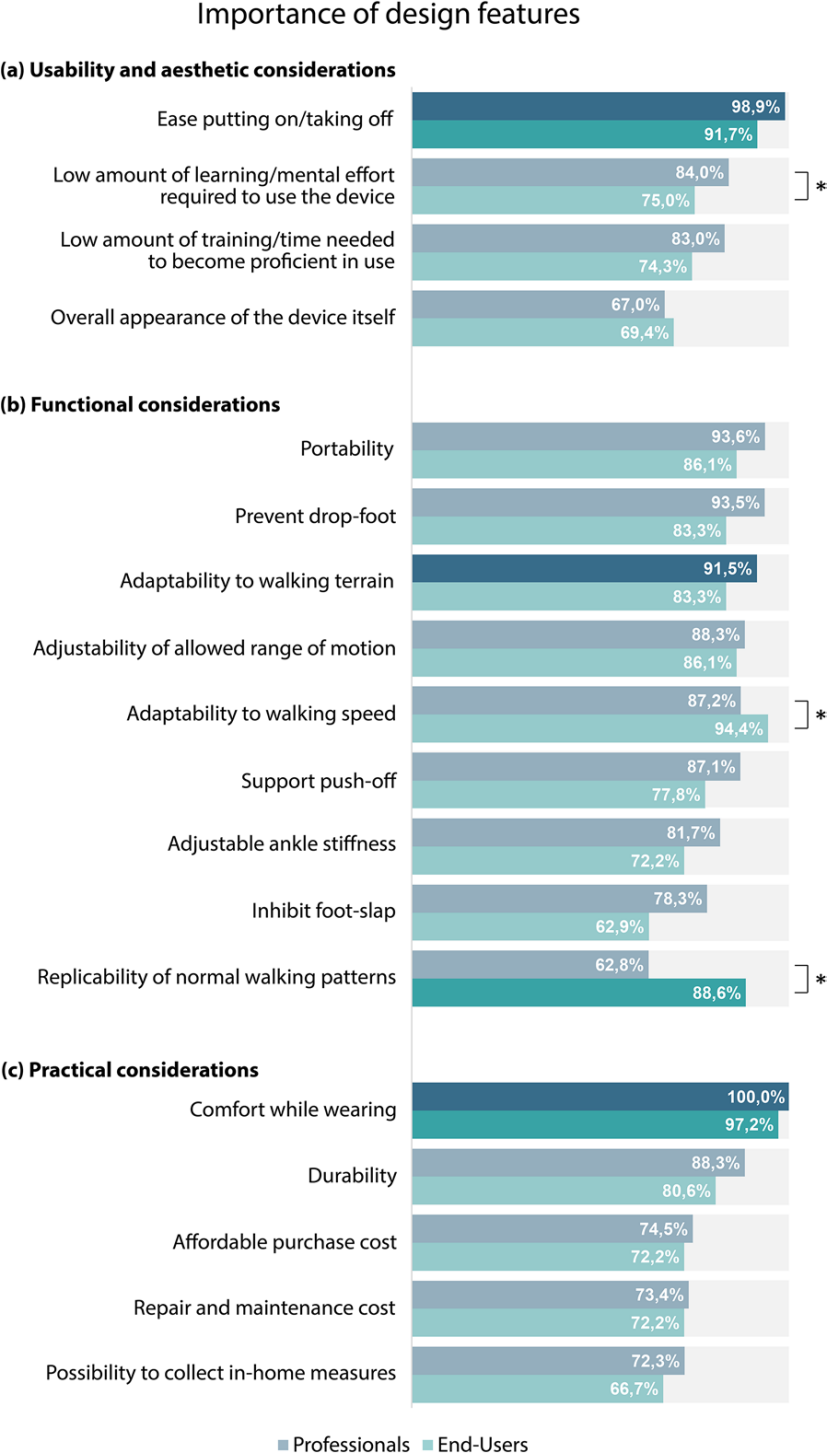
Percentage of respondents who ranked the design features as 4–important or 5–very important. Significant differences between G$_P$ and G$_U$ reported by the Mann–Whitney U tests are marked with (*). The darker bars represent those features that were selected as the top priority by the respondents within each category (**a**–**c**)

Both stakeholder groups (G$_P$ and G$_U$) rated 18 design features on a Likert scale from 1—very unimportant to 5—very important. All features were rated to be important for the majority of all respondents (i.e. >60% of respondents from both groups, Fig. 2). “Ease of putting-on/taking-off” and “comfort while wearing” were rated as ‘important’ or ‘very important’ by more than 90% of respondents from both stakeholder groups.

G$_P$ and G$_U$ agreed on their top priority for usability and aesthetic considerations and for practical considerations (Fig. 2). However, there was no consensus for functional considerations: professionals selected “adaptability to walking terrain” as their top priority, while end-users preferred “replicability of normal walking patterns”.

The Mann–Whitney U tests reported significant differences between the perceived importance for each stakeholder group for three features: end-users’ perceived importance was significantly larger than professionals’ for “replicability of normal walking patterns” (U = 5.52e03, p = 0.001, Median$_{G_P}$ = 4, Median$_{G_U}$ = 5) and “adaptability to walking speed” (U = 5676, p = 0.006, Median$_{G_P}$ = 4, Median$_{G_U}$ = 5). Contrarily, end-users perceived the feature “low amount of learning/mental effort required to use the device” less important than professionals (U = 6619, p < 0.01, Median$_{G_P}$ = 5, Median$_{G_U}$ = 4). For the rest of the features we did not find significant differences, but overall professionals rated the features as more important than end-users did (15 out of 18 features).

#### Type of walking assistance

**Fig. 3:**
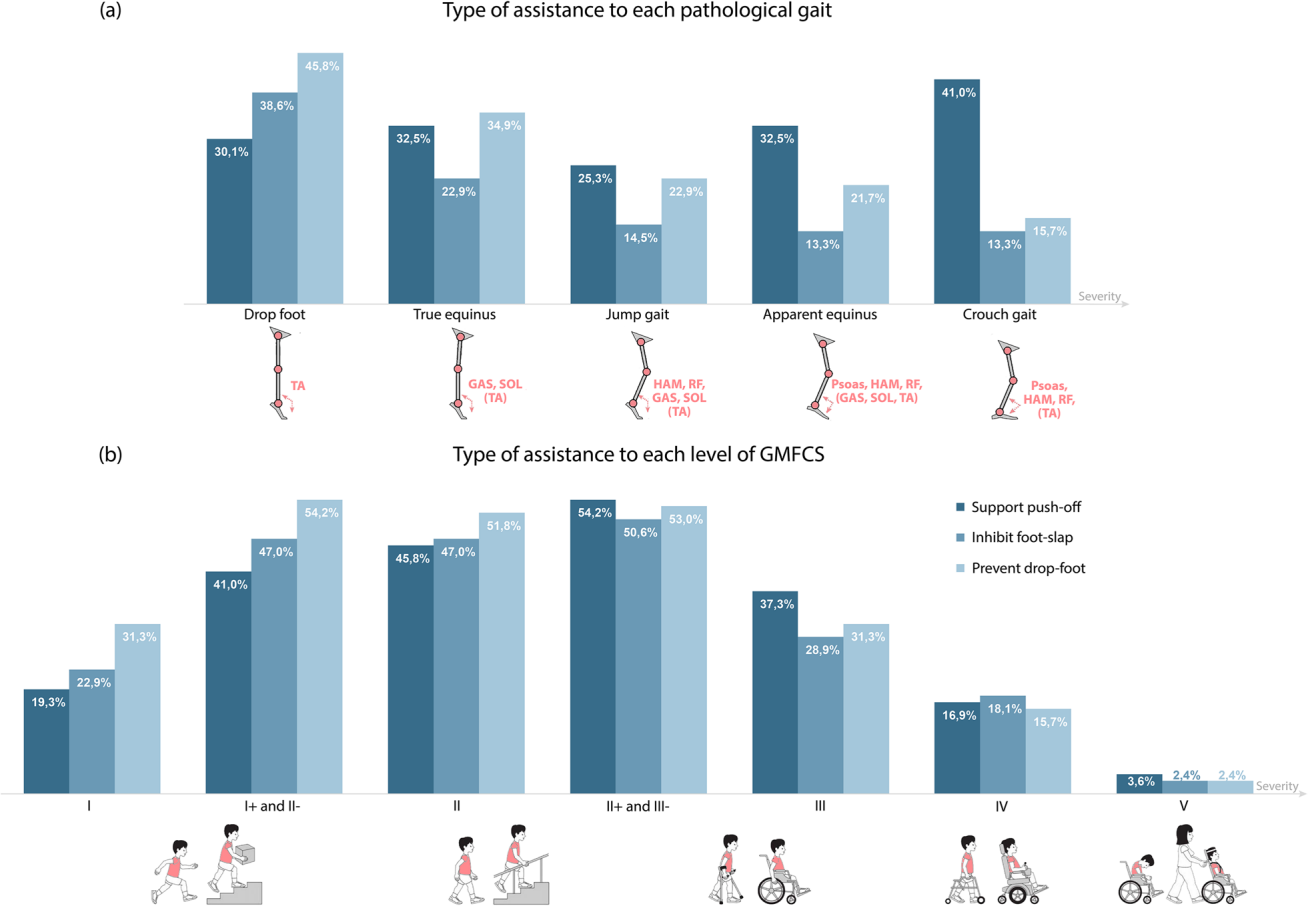
health care professional (83/94) responses to the type of assistance (support push-off in dark blue, inhibit foot-slap in mid blue, or prevent drop-foot in light blue) that would be beneficial depending on **a** patient’s pathological gait and **b** the level of the GMFCS. Multiple response was allowed for this question. TA: tibialis anterior, SOL: soleus, GAS: gastrocnemius, HAMS: hamstrings, RF: rectus femoris

Health care professionals answered two additional questions to identify the type of ankle assistance that should be applied depending on the pathological gait and level of GMFCS (Fig. 3). In their responses they considered that the assistance to prevent drop-foot has a greater benefit for cases of CP that are less severe, while the assistance in push-off becomes more important with more severe gait patterns as apparent equinus or crouch gait (Fig. 3a). Besides, the patients that would benefit the most from the three types of support evaluated are those classified within levels I+ and III- of the GMFCS (Fig. 3b).

### End-users’ expectation for a new device

**Fig. 4:**
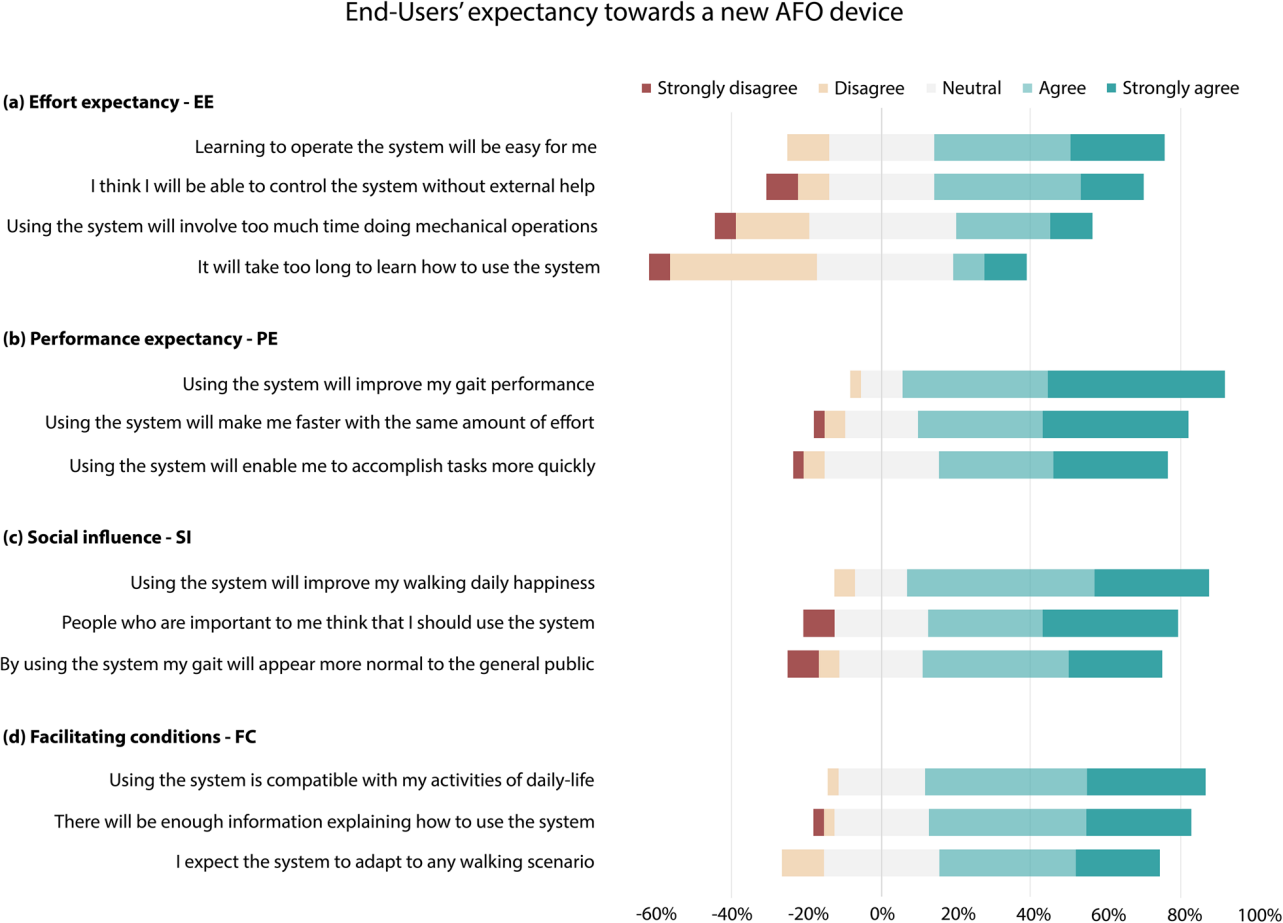
Expectancy of end-Users with CP for a new AFO design. Respondents evaluated different statements on a Likert scale from 1—strongly disagree to 5—strongly agree. Statements were part of four constructs of the (adapted) UTAUT: EE, PE, SI, and FC

The overall perception and expectancy of G$_U$ for a new device is presented in Fig. 4 for the four constructs of the (adapted) UTAUT [32]. The end-users’ acceptance to adopt and use a new system was mostly positive. Although it is expected that some effort will be required to operate the system, the effort is worth perceived as the users expect that the device will improve their gait performance and social influence: averaged percentages of acceptance (i.e. ‘agree’ or ‘strongly agree’) for each construct were 46.53%—EE, 73.15%—PE, 70.27%—SI and 67.35%—FC. Note that for the quantification of averaged percentages of acceptance, negative statements like “it will take too long to learn how to use the system” were reversely counted.

### Prioritized in-home metrics

Descriptive statistics were used to represent the G$_P$’s rated importance for each potential metric to assess patients’ performance in daily-life (Table 3). The most important selected features (top-1) of each category were the gait asymmetry, the stance duration, the angle between foot and shank at heel strike, and the minimum toe clearance at mid-swing. Besides the top selected features, more than 70% of respondents also considered ‘important’ or ‘very important’ the factors of cadence, stride length, gait speed, and the foot-shank angle at both toe-off and mid-stance (Table 3).

**Table 3:** Descriptive statistics to illustrate the rating importance of relevant in-home metrics given by Professionals in CP

Metrics	Mean	SD	Percent importance
General parameters			
Cycle duration	3.8	1.0	63.4
Cadence	4.0	0.8	76.1
Stride length	4.1	0.7	79.4
Stride velocity	3.9	0.8	65.6
**Asymmetry**	**4.0**	**0.9**	**72.3**
Gait speed	4.1	0.8	81.7
Temporal parameters			
**Stance duration**	**4.1**	**0.9**	**77.4**
Swing duration	3.9	0.9	68.5
Double support	3.9	0.9	67.7
Loading dur.	3.8	1.0	63.4
Foot flat dur.	3.8	1.0	62.4
Push-off dur.	3.9	1.0	69.2
Spatial parameters			
Peak angular vel.	3.7	0.9	64.0
Swing speed	3.7	0.9	60.7
**Strike angle (fs)**	**4.3**	**0.9**	**83.3**
Toe-off angle (fs)	4.1	1.0	73.3
Mid stance angle (fs)	4.0	0.9	77.8
Strike angle (fg)	3.9	1.0	67.0
Toe-off angle (fg)	3.8	1.0	61.4
Mid stance angle (fg)	3.8	1.0	68.5
Peak circumduction	3.9	0.9	68.1
Ground clearance			
Max. heel clear.	3.3	1.1	46.2
Max. toe clear. (ms)	3.6	1.1	55.0
**Min. toe clear. (ms)**	**4.1**	**0.9**	**74.7**
Toe clear. (hs)	3.7	1.1	58.9
${\bf n}_{{\bf G}_{\bf P}}$ = **94**			

Bold features represent the ones selected as the top priority by the respondents for each category. The percent of importance includes 4—important + 5—very important

fs: foot-shank; fg: foot-ground; ms: mid-swing; hs: heel strike

### Qualitative analysis of open-ended questions

The 87.2% of G$_P$ and the 80.6% of G$_U$ provided relevant answers to the OE1. These percentages were 63.8% G$_P$ and 47.2% G$_U$ for OE2, and 74.5% G$_P$ and 61.1% G$_U$ for OE3. A complete overview is presented in Appendix B, Table 5.

#### OE1: Daily-life activities that would benefit from an improved gait performance

The theme *General mobility* was the foremost mentioned (68.5% of respondents, 70.7% G$_P$ and 62.1% G$_U$), encompassing subcategories such as Walking (48.6%), Stairs (12.6%) and Running (9.9%).

The second most frequent theme was *Leisure*, with a response rate of 39.6% (45.1% G$_P$, 24.1% G$_U$). It encompassed Play (25.2%), Sports (16.2%), and all other activities associated with free time that require full body motor function.

Other identified themes indicated the importance of having a functional gait pattern in specific locations such as *School* (31.5%), *Non-standardized Terrains* (18.9%) and *Home* (16.2%).

Finally, the theme *Equal social interaction* (20.7%) was identified related to the ability to keep up with able bodied peers and family members.

#### OE2: Limitations of powered exoskeletons for daily-life use

The first theme and main identified problem of powered exoskeletons was their *Bulkiness* (45.5% respondents, 45% G$_P$, 47.1% G$_U$), including subcategories as Weight (31.2%) and Volume (27.3%). The second most frequent theme was *User friendliness* (39.0%), followed by *Cost* (29.9%), *Control* (28.6%), and *Adaptability* (20.8%) of the device.

Themes with lower frequencies were *Availability* (11.7%), understood as the possibility of getting access to an exoskeleton, *Flexibility & ROM* (7.8%), *Acceptance* (6.5%) and *Durability* (5.2%), the last two only mentioned by the group of professionals (Appendix B).

#### OE3: Limitations of passive AFOs for daily-life use

The most important problem of current AFOs and principal theme based on G$_U$ was the lack of *Comfort* (21.4% G$_P$, **50%** G$_U$). In the case of G$_P$, the predominant limitation of current AFOs is the *Adaptability* of these devices (**55.7%** G$_P$, 36.4% G$_U$) to both patients’ needs (i.e. type and level of assistance) and environment (i.e. type of walking surface).

Other identified themes were *Flexibility & ROM* (22.7%), *Bulkiness* (17.4%), *Wearability* (9.8%), specifically referring to the difficulties to combine AFOs with clothing and shoes, *Metrics* (9.8%), with answers like “Possibility to test different AFO models with quantitative metrics to evaluate which solution is the best for a specific patient”, *User friendliness* (9.8%), *Cost* (8.7%), *Durability* (6.5%) and the improvement of general *Walking* (5.4%). In the latter, professionals highlighted the necessity to improve ‘Functional’ Walking, while end-users stressed the desire of generating ‘Normal’ Walking patterns with AFOs (Appendix B).

The necessity of new AFOs that reduce *Energy cost* was a theme mentioned by 20% of professionals, but not by the end-users group.

## Discussion

### Summary of the results

The present study was designed to collect the existing limitations on the AFO technology for individuals with CP and identify areas of improvement. The results derived from the surveys provide insights on the stakeholders’ needs and criteria to assist pathological gait in CP, highlighting important required features that may be useful for both (1) the assessment and prescription of current AFOs, and (2) the development of future novel devices. A recent publication [21] has previously assessed some of our concerns and strengthen the pertinent development of new research in this field. However, Zaino et al. [21] only performed a qualitative analysis, while we provide both quantitative and qualitative analysis and include other clusters that were not considered in [21] (e.g. *Gait management and AFO prescription* and *Relevant in-home metrics*).

In total, 94 professionals in CP (83 of them working within the health care sector) and 36 end-users with CP (patients or families) responded to our surveys. We acknowledge that some individuals with CP who answered the questionnaire and who are affected by severe motor impairments (i.e. GMFCS V) are maybe unlikely to benefit from assistive AFOs to promote their walking capabilities in daily-life. However, we also gathered their responses and recognized their wishes as they all have residual functions to contribute with small actions to everyday tasks.

The most valuable feature identified by both stakeholder groups in the close-ended questions was the “comfort while wearing” the assistive device, i.e. avoiding skin pressure, friction or abrasions. This has also been a primary concern of previous studies about the efficacy of AFOs for CP [13, 21], and other studies focused on assistive technology for patients with spinal cord injury or stroke [30]. The second most valuable design feature highlighted by both stakeholder groups was the “ease of putting-on and taking-off” the device. These impressions on the comfort and usability of the device were also reflected in the open-ended questions, where stakeholders proposed a change to have a more breathable and softer AFO material and a better fit to the child’s foot. It is remarkable for us that even considering that “costs for replacement or maintenance” can be substantial during childhood and adolescent growth, the rated importance for these cost features did not stand out compared to the comfort and usability of the device.

All design features of the survey were considered to be important, but generally professionals rated them higher than end-users did (Fig. 2). However, a clear deviation of this pattern was observed for the “replicability of normal walking patterns”: 62.8% G$_P$ vs 88.6% $G_U$ considered this feature to be ‘important’ or ‘very important’. This explicit divergence made clear that both stakeholder groups differed when selecting their top priority under functional considerations (Fig. 2b): while professionals preferred to provide the child with higher autonomy by making the AFO “adaptable to different walking terrains”, end-users made their preference for having a “more normal walking pattern”. The reason behind this might be that professionals were considering the improvements on functional gait levels, but end-users were thinking more about social acceptance and participation at task levels.

The aforementioned rating of functional design features seemed to result in an apparent inconsistency, as the highest percentage of importance rating does not correspond to the top-1 priority selected by any stakeholder group (although it was in their top-2 or top-3). A similar circumstance happened when selecting the top-1 general parameter under *Relevant in-home metrics* (Table 3). The explanation for this might be that respondents sometimes scored features with 4—important or 5—very important indistinctly, and they only selected their real first preference when being asked for their top priorities among the scored ones. That confirms the importance of including a question for prioritizing the rated features in the surveys.

Concerning the classification that professionals made of the type of walking assistance with respect to the level of the GMFCS, there were some clear trends (Fig. 3): (1) patients classified as GMFCS I normally have the ability to walk, so minimal additional assistance of any type is needed in those cases; (2) patients classified within levels GMFCS IV and V can barely walk, so they do not benefit that much from the assistances conceived here; and (3) patients classified within GMFCS I+ to III are the ones that can benefit the most from the extra support provided by dynamic AFOs. In these cases, the prevention of drop-foot is more important for less severely affected gait patterns (GMFCS I+ and II), while the push-off support becomes more important as gait patterns get more severely affected (GMFCS II+ and III). However, each level of GMFCS involves heterogeneous patient’s behaviours [22], and as such, the relationship between Fig. 3a and b is not direct nor trivial. Although there is an overlap (i.e. drop-foot gait is mostly seen in GMFCS I and crouch gait in GMFCS III), children classified between levels I+ and III can exhibit any type of the pathological gait patterns presented.

This convoluted connection between the level of the GMFCS, the patient’s pathological gait and the type of assistance needed makes it hard to prescribe the most suitable AFO for a specific patient [13, 18]. This fact was also expressed in the cluster *Gait management and AFO prescription* of the survey, where health care professionals reported a lack of confidence concerning decision-making about AFO prescription. This might (also) be related to the fact that more than 79% of health care professionals thought that the performance of patients in the laboratory (e.g. gait analysis) is different than in real-life settings. This behaviour has been a long investigation of psychologists and it is known as the ‘Hawthorne effect’ [33], which states that humans act differently if they think they are being observed. The Hawthorne effect has previously been verified in children with CP [20, 22], suggesting that their walking *capacity* demonstrated in a standardized environment (laboratory) is usually overestimated and exceeds their walking *performance* in real-life settings. That is likely the reason why around 98% of the health care professionals emphasized the importance of gathering information regarding the use of assistive technology at home, as this may enrich the assessments and evaluations at the clinic. Table 3 gave an overview in this regard, about the importance given by the professionals in CP for the principal in-home metrics to be collected. These in-home metrics would be useful not only to assess the patient’s performance, but also to evaluate different AFO models using quantitative parameters, as it was stated by the respondents in the OE questions.

### Implications for future devices

New and effective approaches are needed to assess and treat CP pathological gait in non-standardized settings [21]. Existing traditional (passive) solutions present an inherent function as mobility devices; however, from the responses to our survey we can extract that they are not adaptable to a specific patient’s needs, nor do they enhance their existing capabilities. Although there have been promising advances (especially with the dynamic ADR-AFOs), they still lack the necessary evidence to demonstrate the adaptability to different scenarios and the achievement of lasting improvements and long-term effects in general gait quality [12–14, 23, 24].

For new AFO designs, the benefits of (powered) robotic technology (e.g. tailoring the assistance provided) should be considered. User’s expectations to adopt a new solution are positive (Fig. 4). However, it is key to put effort in addressing the current problems of powered devices identified by the respondents of this survey (see Appendix B), including the comfort, weight, bulkiness, safety, operability and user-friendliness. These are crucial factors for the implementation of assistive devices in daily-life, and are also related to the features highlighted in a recent article [21, 34] for the adoption of robotic technology for pediatric rehabilitation. Moreover, to satisfy the requirements of both health care professionals and end-users, there should be a trade-off between the improvement of functional levels, the provision of higher autonomy, and the social acceptance while using the device.

Finally, the incorporation of sensors within the design of new AFOs might be an option to provide metrics and assess the user’s gait in daily-life to inform clinical decision-making.

### Study limitations

The survey results encompassed responses of stakeholders from ten different countries. However, the majority of these stakeholders were from Spain and The Netherlands. This might provoke some bias with the representation of the broader world population. A second bias might come from the voluntary nature of participation in the online surveys, which might cause that some questions are responded positively towards the necessity of changes in current gait assistive technology for CP [35]. Finally, although we included the option “other” in most of the clusters of our surveys allowing the respondents to answer information different than the pre-selected one, respondents normally opted for just scoring the pre-selected options.

## Conclusion

This exploratory study provides insights into the weighted desires of children with CP, their families and professionals in the field towards the use and design of (AFO) assistive devices. The study suggests that the identified prioritized areas of improvement should be considered as important information for new designs of these assistive devices, but it is not meant to be a resolute guide, nor does it attempt to rationale for the biomechanical basis to influence in gait performance.

With this research we tried to give clarity on (1) what needs to be improved in current assistive technology to enrich gait in daily-life activities, and (2) what type of day-to-day performance measurements may allow better personalization of gait management and AFO prescription. The outcomes of our investigation bring different and complementary information, which is valuable for both designers of assistive devices for CP and clinicians involved in treatment and follow-up care of these patients.

### Supplementary Information

**Additional file 1.** Final version of the English survey for Professionals stakeholder group (*G*_*P*_).

**Additional file 2. **Final version of the English survey for end-users with CP and families (*G*_*U*_).

## Data Availability

The anonymized datasets generated and/or analyzed during the current study are available from the corresponding author on reasonable request.

## Appendix A

In the following table we report a relationship between the most predominant pathological gaits in CP and the primary management methods currently applied. The traditional AFOs considered for orthotic management are:

- Ground reaction AFO (GRAFO): rigid orthosis with a ventral shell that blocks any movement of the anatomical ankle joint in the interest of enabling knee extension in terminal stance
- Solid AFO (SAFO): rigid orthosis covering the foot and the shank with a dorsal shell that blocks any movement of the anatomical ankle joint
- Hinged AFO (HAFO): orthosis with a dorsal shell that blocks any plantarflexion but enables dorsiflexion with a defined pivot point in the anatomical ankle joint. It does not have a spring effect nor a dorsiflexion stop
- Posterior leaf-spring AFO (PLS-AFO): orthosis with a leaf spring behind the Achilles tendon. It provides flexibility at the ankle joint and allows passive ankle plantar- dorsiflexion during the stance phase. It also corrects excessive platarflexion during swing
- Supramalleolar AFO (SPM-AFO): used to increase ankle medio-lateral stability and foot alignment while allowing full ankle plantar-dorsiflexion

**Table 4:** Relationship between pathological gait types and primary management methods applied

Pathological gait	Representation and muscles	Characteristics	Prevalence	Orthotic management*	AFO representation	Other management
Drop foot		Drop foot during swing due to inability to control ankle dorsiflexors. No calf contracture, so during stance, dorsiflexion is normal. Lack of first rocker	Rare. Normally it progresses to others more severe	HAFO or PLS-AFO		Not applicable
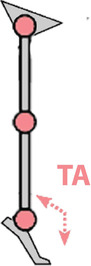
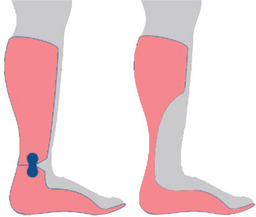
True equinus (w/o knee recurvatum)		True equinus during stance due to spasticity or contracture of the gastroc-soleus muscles. Drop foot in swing for impaired function in ankle dorsiflexors	Very common	HAFO or PLS-AFO		BTX-A to calf, Tendo Achilles and/or calf lengthening
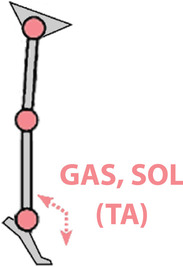
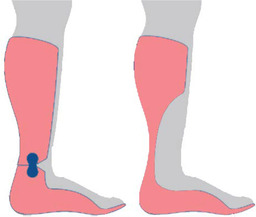
Jump gait (w/o stiff knee)		Spasticity on hamstrings and hip flexors in addition to calf spasticity/contracture. The ankle is in equinus, with knee and hip in flexion and anterior pelvis tilt	Very common	HAFO, PLS-AFO or SAFO		BTX-A to calf and hamstrings. SEMLS for addressing lever arm dysfunction
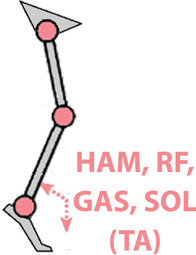
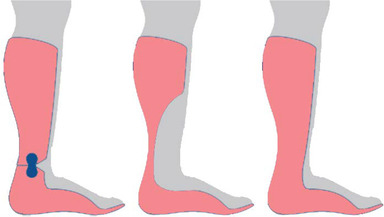
Apparent equinus (w/o stiff knee)		Progression of pathological gait with child’s growth. Ankle has an apparent normal dorsiflexion during stance, but knee and hip are in excessive flexion.	Common	PLS-AFO, SAFO or GRAFO		No BTX-A to the calf, as it would cause crouch gait. SEMLS for addressing lever arm dysfunction
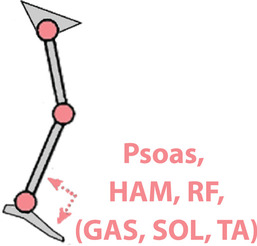
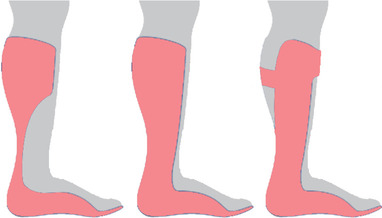
Crouch gait		Excessive ankle dorsiflexion during stance in combination with excessive knee and hip flexion.	Only severe cases	GRAFO		The pathology is normally too advanced to use BTX-A, although if the child is young, BTX-A can be used on HAMS and hip flexors. SEMLS for addressing lever arm dysfunction
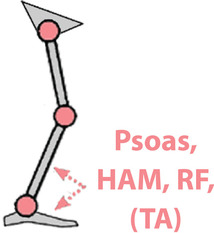
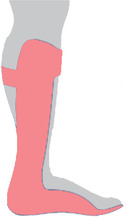

*Choice according to the PF-KE couple [36] and other parameters [14]. BTX-A: Botulinum toxin type A; SEMLS: single-event multilevel surgery; TA: tibialis anterior; SOL: soleus; GAS: gastrocnemius; HAMS: hamstrings; RF: rectus femoris

## Appendix B

Themes overview derived from responses to the OE questions is presented here.

**Table 5:** Qualitative themes from the content analysis

Theme	Associated categories	Percent		
		$G_P$	$G_U$	Total
OE1: Daily-life activities that would benefit from an improved gait performance				
*“Both indoors and outdoors ambulation”, “Playing with friends”, “Going to school”, “Adjusting to changing circumstances outside”, “Keep friends’ velocity during walking”, “To walk normally over all kind of terrains”, “To be independent for social activities”*				
*General mobility*	Walking, stairs, running, jumping	70.7%	62.1%	68.5%
*Leisure*	Play, sports	45.1%	24.1%	39.6%
*School*	Mobility at school	35.4%	20.7%	31.5%
*Equal social interaction*	Keep up with able bodied peers	25.6%	6.9%	20.7%
*Non-standard. terrains*	Parks, playgrounds, nature	19.5%	17.2%	18.9%
*Home*	Mobility between and inside home rooms	18.3%	10.3%	16.2%
*Other*	–	2.4%	0.0%	1.8%
OE2: Limitations of powered exoskeletons for daily-life use				
*“They are quite big and bulky”, “They should be easier of putting on/taking off”, “Adaptability and ajustability”, “Affordable cost and accessibility”, “More compact”, “More functionality aimed for the wishes of the individual”, “Batteries”*				
*Bulkiness*	Weight, volume	45.0%	47.1%	45.5%
*User friendliness*	Ease of use	41.7%	29.4%	39.0%
*Cost*	Purchase and reparation costs	33.3%	17.6%	29.9%
*Control*	Control requirements and manipulation	31.7%	17.6%	28.6%
*Adaptability*	Patient’s needs, environment	23.3%	11.8%	20.8%
*Availability*	Getting access to its use	11.7%	11.8%	11.7%
*Flexibility and ROM*	Possibility of movements, compliance	6.7%	11.8%	7.8%
*Acceptance*	Approval by end-user	8.3%	0.0%	6.5%
*Durability*	Lifetime	6.7%	0.0%	5.2%
*Other*	–	8.3%	11.8%	9.1%
OE3: Limitations of passive AFOs for daily-life use				
*“AFOs impede activities like climbing stairs or jumping”, “Adjustment to foot size and breathability”, “Don’t properly support push-off”, “Different support depending on the patient’s needs”, “Cost and comfort”, “They are too rigid and uncomfortable”*				
*Adaptability*	Patient’s needs, Environment	55.7%	36.4%	51.1%
*Flexibility and ROM*	Possibility of movements, compliance	31.4%	22.7%	29.3%
*Comfort*	Avoid pressure, friction, abrasions	21.4%	50.0%	28.3%
*Bulkiness*	Wearability, weight, volume	12.9%	31.8%	17.4%
*Energy cost*	–	20.0%	0.0%	15.2%
*Metrics*	Possibility of assessment while wearing	11.4%	4.5%	9.8%
*User friendliness*	Ease of use	10.0%	9.1%	9.8%
*Cost*	Purchase and reparation costs	7.1%	13.6%	8.7%
*Durability*	Lifetime	7.1%	4.5%	6.5%
*Walking*	Walking normal, functional	4.3%	9.1%	5.4%
*Other*	–	10.0%	13.6%	10.9%

Frequency of mentioning for G$_P$, G$_U$ and total normalized participation. Examples of literal responses are between quotation marks

## Abbreviations

CP: Cerebral Palsy
AFO: Ankle-foot orthosis
GMFCS: Gross motor function classification system
GRAFO: Ground reaction AFO
SAFO: Solid AFO
HAFO: Hinged AFO
PLS-AFO: Posterior leaf-spring AFO
SMP-AFO: Supramalleolar AFO
ADR-AFO: Adjustable dynamic response AFO
ROM: Range of motion
G$_P$: Group of professionals in CP responding the survey
G$_U$: Group of end-users (children with CP and families) responding the survey
OE: Open-ended
CE: Closed-ended
UTAUT: Unified Theory of Acceptance and Use of Technology questionnaire
EE: Effort expectancy
PE: Performance expectancy
SI: Social influence
FC: Facilitating conditions
BTX-A: Botulinum toxin type A
SEMLS: Single-event multilevel surgery
TA: Tibialis anterior SOL soleus
GAS: Gastrocnemius
HAMS: Hamstrings
RF: Rectus femoris
PF-KE: Plantarflexion-knee extension couple

## Footnotes

1 Gillette Children’s hospital and Shirley Ryan Ability Lab (United States of America), Roessingh Research and Development, Sint Maartenskliniek and Amsterdam UMC (The Netherlands), Hospital Niño Jesús (Spain)
