# Supplementary material for: Perspectives on ankle-foot technology for improving gait performance of children with Cerebral Palsy in daily-life: requirements, needs and wishes
Source: J Neuroeng Rehabil. 2023 Apr 12;20:44. doi: 10.1186/s12984-023-01162-3 (PMC10099972; doi:10.1186/s12984-023-01162-3)
Supplement: Supplementary file 1 — Additional file 1. Final version of the English survey for Professionals stakeholder group (GP). [file 12984_2023_1162_MOESM1_ESM.pdf]

inGAIT

Questionnaire for professionals

Over recent years, traditional (rigid or hinged) orthotic bracing has been complemented with innovative technological solutions such as powered exoskeletons and adjustable dynamic response (ADR) orthosis. However, there are practical barriers that prevent the extensive use of these devices on daily-life activities. On one hand, powered devices are not ready to be used out of controlled environments due to their bulkiness, lack of user-friendliness and weight. On the other hand, current passive orthotic bracing solutions may prevent acceleration/deceleration during walking, block or restrict motion and provide assistance that is not effectively timed. Moreover, none of the existing devices can adapt their support to the large variety of environments and conditions of daily-living.

With inGAIT project, we aim to further understand the requirements for improving walking performance of children with cerebral palsy out of controlled environments. The results of this research will provide significant scientific knowledge to allow an upgrade of future orthosis designs and a step forward towards the assessment of patients' performance through in-home metrics reports.

We appreciate your completion of the following questionnaire. Your answers will be made anonymous, kept confidential and only used for the purpose of inGAIT research project. The security measures of the University of Twente research storage will apply. Personal data will only include general information, such as age, sex or country of residence. No sensitive personal data will be collected.

☐ I am aware that my participation to the questionnaire is voluntary and I am willing to provice my answers

1. Demographics

1.1. What is your sex?

- ○ Male
- ○ Female
- ○ Other
- ○ Prefer not to disclose

1.2. What is your age?

- ○ Under 18
- ○ 18-24
- ○ 25-34
- ○ 35-44
- ○ 45-54
- ○ 55-64
- ○ 65 or above
- ○ Prefer not to answer

1.3. What country do you currently live in?

____

1.4. What is your current profession?

- ○ Equipment Vendor
- ○ Researcher
- ○ Surgeon
- ○ Orthotist
- ○ Rehabilitation Physician
- ○ Physiotherapist
- ○ Other (please specify) ________________________

1.5. How long have you worked in your field?

- ○ Under 1 year
- ○ 1-4 years
- ○ 5-10 years
- ○ 11+ years

1.6. Do you have hands-on experience with orthosis for Cerebral Palsy (CP)?

- ○ Yes
- ○ No

1.7. Have you ever worked with or used a powered exoskeleton or an adjustable-reactive response (ADR) AFO?

- ○ Yes
- ○ No

2. Gait management and AFO prescription

How much do you agree or disagree with the following statements. ONLY ANSWER IF YOU ARE A HEALTH CARE PROFESSIONAL. OTHERWISE, SKIP TO SECTION 3.

	Strongly disagree	Disagree	Neutral	Agree	Strongly agree
There is enough information to feel confident when prescribing the correct AFO type (solid, hinged, ADR…) for a specific patient	○	○	○	○	○
I think patient's performance in clinic is different than in real-life settings	○	○	○	○	○
A report on the use of AFO technology on daily-life could provide useful information to improve the assessment in clinic	○	○	○	○	○
It would be important to get information about walking on daily-life activities of children with CP	○	○	○	○	○

3. Importance of support devices design features

How important you consider the following features for an ideal home-use technology to improve walking abilities

3.1. Usability and aesthetic considerations

	Very unimportant	Unimportant	Neutral	Important	Very important
Ease of putting on/taking off	○	○	○	○	○
Low amount of learning/mental effort required to use the device	○	○	○	○	○
Low amount of training/time needed to become proficient in use	○	○	○	○	○
Overall appearance of the device itself	○	○	○	○	○
Other (specify):	○	○	○	○	○

It might be that you have selected ''important'' or ''very important'' in several previous categories. Please select among all of them the TOP-3, in order of importance:

Selection 1	Selection 2	Selection 3

3.2. Functional considerations

	Very unimportant	Unimportant	Neutral	Important	Very important
Adaptability to walking speed	○	○	○	○	○
Adaptability to walking terrain (stair, ramp, uneven surface…)	○	○	○	○	○
Replicability of normal walking patterns	○	○	○	○	○
Portability (weight, shape…)	○	○	○	○	○
Adjustability of allowed range of motion	○	○	○	○	○
Adjustable ankle stiffness	○	○	○	○	○
Support push-off*	○	○	○	○	○
Inhibit foot slap*	○	○	○	○	○
Prevent drop-foot*	○	○	○	○	○
Other (specify):	○	○	○	○	○

Of the categories listed, which three are the most important? Please select among all of them the TOP-3, in order of importance:

Selection 1	Selection 2	Selection 3

*If you selected "Very Important" or "Important" for the marked (*) features, please indicate to which pathological gait and level of GMFCS is most applicable (select all that correspond). ONLY ANSWER IF YOU ARE A HEALTH CARE PROFESSIONAL. OTHERWISE, SKIP TO SECTION 3.3:

Support push-off

☐	☐	☐	☐	☐
Drop foot	True equinus (w/o knee recurvatum)	Jump gait (w/o stiff knee)	Apparent equinus (w/o stiff knee)	Crouch gait (w/o stiff knee)

☐	☐	☐	☐	☐	☐	☐
GMFCS I	GMFCS I+ II-	GMFCS II	GMFCS II+ III-	GMFCS III	GMFCS IV	GMFCS V

Inhibit foot slap

☐	☐	☐	☐	☐
Drop foot	True equinus w/o knee recurvatum	Jump gait (w/o stiff knee)	Apparent equinus (w/o stiff knee)	Crouch gait (w/o stiff knee)

☐	☐	☐	☐	☐	☐	☐
GMFCS I	GMFCS I+ II-	GMFCS II	GMFCS II+ III-	GMFCS III	GMFCS IV	GMFCS V

Prevent drop-foot				
☐ Drop foot	☐ True equinus w/o knee recurvatum	☐ Jump gait (w/o stiff knee)	☐ Apparent equinus (w/o stiff knee)	☐ Crouch gait (w/o stiff knee)

☐ GMFCS I	☐ GMFCS I+ II-	☐ GMFCS II	☐ GMFCS II+ III-	☐ GMFCS III	☐ GMFCS IV	☐ GMFCS V

3.3. Practical considerations

	Very unimportant	Unimportant	Neutral	Important	Very important
Affordable purchase cost	○	○	○	○	○
Repair and maintenance cost	○	○	○	○	○
Comfort while wearing	○	○	○	○	○
Possibility to collect in-home measures to improve the post analysis in clinic	○	○	○	○	○
Durability	○	○	○	○	○
Other (specify):	○	○	○	○	○

Of the categories listed, which three are most important? Please select among all of them the TOP-3, in order of importance:

Selection 1	Selection 2	Selection 3

4. Relevant in-home metrics

How important you consider the following features as possible metrics to record at home with the aim of improving a posterior assessment in clinic?. All metrics are focused on information at ankle level and would include several statistics (mean, standard deviation, maximum, minimum…)

4.1. General parameters

		Very unimportant	Unimportant	Neutral	Important	Very important
Cycle duration	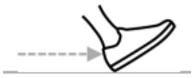	○	○	○	○	○
Cadence	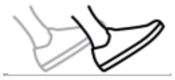	○	○	○	○	○
Stride length	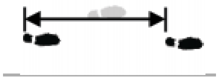	○	○	○	○	○

Stride velocity	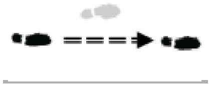	○	○	○	○	○
Asymmetry	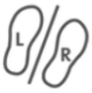	○	○	○	○	○
Gait speed	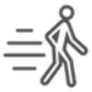	○	○	○	○	○
Other (specify):		○	○	○	○	○

Of the categories listed, which three are most important? Please select among all of them the TOP-3, in order of importance:

Selection 1 Selection 2 Selection 3

4.2. Temporal parameters

		Very unimportant	Unimportant	Neutral	Important	Very important
Stance duration	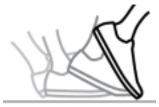	○	○	○	○	○
Swing duration	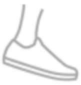	○	○	○	○	○
Double support duration	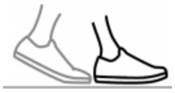	○	○	○	○	○
Loading duration	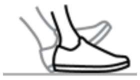	○	○	○	○	○
Foot-flat duration (mid stance)	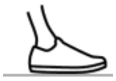	○	○	○	○	○
Push-off duration	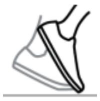	○	○	○	○	○
Other (specify):		○	○	○	○	○

Of the categories listed, which three are most important? Please select among all of them the TOP-3, in order of importance:

Selection 1 Selection 2 Selection 3

4.3. Spatial parameters

		Very unimportant	Unimportant	Neutral	Important	Very important
Peak angular velocity	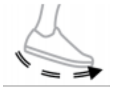	○	○	○	○	○
Swing speed	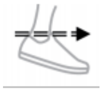	○	○	○	○	○
Strike angle (foot-shank)	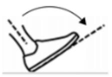	○	○	○	○	○
Toe-off angle (foot-shank)	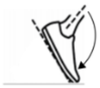	○	○	○	○	○
Mid stance angle (foot-shank)		○	○	○	○	○
Strike angle (foot-ground)	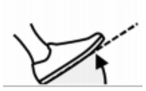	○	○	○	○	○
Toe-off angle (foot-ground)	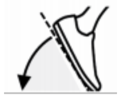	○	○	○	○	○
Mid stance angle (foot-ground)		○	○	○	○	○
Peak swing width circumduction	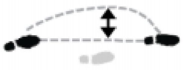	○	○	○	○	○
Other (specify):		○	○	○	○	○

Of the categories listed, which three are most important? Please select among all of them the TOP-3, in order of importance:

Selection 1 Selection 2 Selection 3

4.4. Ground clearance parameters

		Very unimportant	Unimportant	Neutral	Important	Very important
Max heel clearance	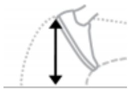	○	○	○	○	○
Max toe clearance mid swing	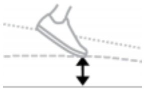	○	○	○	○	○
Min toe clearance mid swing	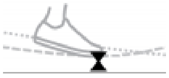	○	○	○	○	○
Toe clearance at heel strike	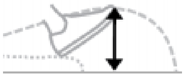	○	○	○	○	○
Other (specify):		○	○	○	○	○

Of the categories listed, which three are most important? Please select among all of them the TOP-3, in order of importance:

Selection 1	Selection 2	Selection 3

5. Importance of gait performance (open-ended)

5.1. Which daily-life activities would benefit from improved gait performance in children with CP?

6. Current problems of support devices to be used in daily-life activities

6.1. What changes to the current exoskeletons are needed to improve gait performance in daily-life situations?

6.2. What changes to the current AFOs are needed to improve gait performance in daily-life situations?
