# Supplementary material for: Perspectives on ankle-foot technology for improving gait performance of children with Cerebral Palsy in daily-life: requirements, needs and wishes
Source: J Neuroeng Rehabil. 2023 Apr 12;20:44. doi: 10.1186/s12984-023-01162-3 (PMC10099972; doi:10.1186/s12984-023-01162-3)

Over recent years, traditional orthotic bracing has expanded to include technological solutions such as powered exoskeletons that relieve muscle burden, and adjustable dynamic response (ADR) orthosis that provide greater flexibility in a brace. However, there are practical barriers that prevent the extensive use of these devices on daily-life activities. On one hand, powered devices are not ready to be used out of controlled environments due to their bulkiness, weight, and difficulty to use. On the other hand, current passive orthotic bracing solutions may limit slowing down or speeding up while walking, restrict motion and do not offer versatile assistance. Moreover, none of the existing devices can adapt their support to the large variety of terrains and conditions of daily-living.

With inGAIT project, we aim to further understand the requirements for improving walking performance of children with cerebral palsy out of controlled environments. The results of this research will provide significant scientific knowledge, allow innovation in future orthosis designs and develop in-home assessment of orthosis performance.

We appreciate your participation in the following questionnaire. Your answers will be made anonymous, kept confidential and only used for the purpose of inGAIT research project. The security measures of the University of Twente research storage will apply. Personal data will only include general information, such as age, sex or country of residence. No sensitive personal data will be collected.

☐ I am aware that my participation to the questionnaire is voluntary and I am willing to provide my answers

1. Demographics

1.1. What is the child’s sex?

○ Male

○ Female

○ Other

○ Prefer not to disclose

1.2. What is the child’s age?

○ Under 3

○ 3 - 7

○ 8 - 12

○ 13 - 17

○ 18 or above

○ Prefer not to Answer

1.3. What country does the child currently live in?

____

1.4. What is the child’s gross motor function level? (see image for reference)

- ○ GMFCS I
- ○ GMFCS II
- ○ GMFCS III
- ○ GMFCS IV
- ○ GMFCS V

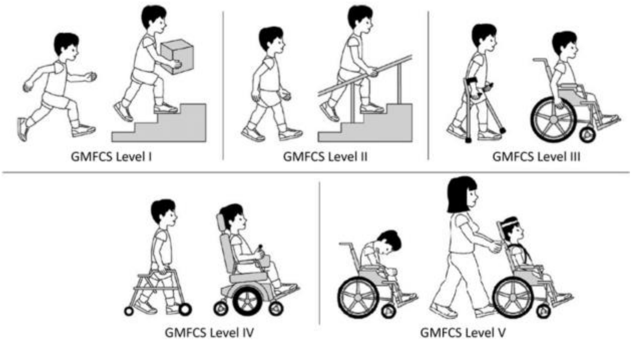

1.5. Has the child ever used a powered exoskeleton or an adjustable-reactive response (ADR) AFO?

- ○ Yes
- ○ No

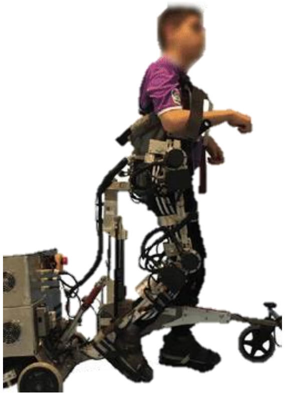

Example of powered exoskeleton

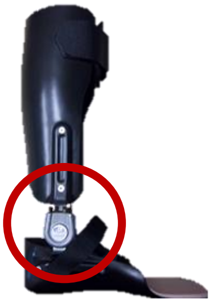

Example of ADR-AFO

1.6. Which type of AFO is the child currently using?

Name	Picture
Supramalleolar AFO	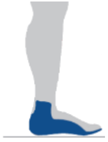
Solid AFO	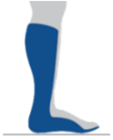
Ground reaction AFO	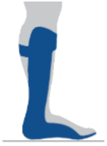
Hinged AFO	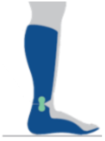
Posterior Leaf spring AFO	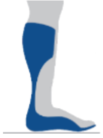
Adjustable dynamic response AFO	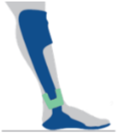

1.7. Where does the child normally use the AFO? (select all which correspond)

- ☐ At home
- ☐ At school
- ☐ For social activities
- ☐ For sports
- ☐ General mobility
- ☐ Other (specify):

2. Importance of support device design features

How important you consider the following features for an ideal home-use technology to improve walking abilities

2.1. Usability and aesthetic considerations

	Very unimportant	Unimportant	Neutral	Important	Very important
Ease of putting on/taking off	○	○	○	○	○
Low amount of learning/mental effort required to use the device	○	○	○	○	○
Low amount of training/time needed for effective use	○	○	○	○	○
Overall appearance of the device itself	○	○	○	○	○
Other (specify):	○	○	○	○	○

It might be that you selected ''important'' or ''very important'' in several previous categories. Please select among all of them the TOP-3, in order of importance:

Selection 1 Selection 2 Selection 3

2.2. Functional considerations

	Very unimportant	Unimportant	Neutral	Important	Very important
Adaptability to walking speed	○	○	○	○	○
Adaptability to walking area (stair, ramp, uneven surface…)	○	○	○	○	○
Replicability of normal walking patterns	○	○	○	○	○
Portability (weight, shape…)	○	○	○	○	○
Adjustability of allowed range of motion	○	○	○	○	○
Adjustable ankle stiffness	○	○	○	○	○
Support push-off	○	○	○	○	○
Inhibit foot slap	○	○	○	○	○
Prevent drop-foot	○	○	○	○	○
Other (specify):	○	○	○	○	○

Of the categories listed, which three are most important? Please select among all of them the TOP-3, in order of importance:

Selection 1 Selection 2 Selection 3

2.3. Practical considerations

	Very unimportant	Unimportant	Neutral	Important	Very important
Affordable purchase cost	○	○	○	○	○
Repair and maintenance cost	○	○	○	○	○
Comfort while wearing	○	○	○	○	○
Possibility to collect in-home measures to improve the post analysis in clinic	○	○	○	○	○
Durability	○	○	○	○	○
Other (specify):	○	○	○	○	○

Of the categories listed, which three are most important? Please select among all of them the TOP-3, in order of importance:

Selection 1	Selection 2	Selection 3

3. Patients' expectations to new ADR-AFO

Imagine you have received a new ADR-AFO prescription as you answer the following questions.

3.1. Effort expectancy

	Strongly disagree	Disagree	Neutral	Agree	Strongly agree
Learning to operate the system would be easy for me	○	○	○	○	○
Using the system will involve too much time doing mechanical operations	○	○	○	○	○
It will take too long to learn how to use the system	○	○	○	○	○
I think I will be able to control the system without external help	○	○	○	○	○

3.2. Performance expectancy

	Strongly disagree	Disagree	Neutral	Agree	Strongly agree
Using the system would enable me to accomplish tasks more quickly	○	○	○	○	○
Using the system would improve my gait performance	○	○	○	○	○
Using the system will make me stronger/faster with the same amount of effort	○	○	○	○	○

3.3. Social influence

	Strongly disagree	Disagree	Neutral	Agree	Strongly agree
People who are important to me think that I should use the system	○	○	○	○	○
Using the system will improve my walking and daily happiness	○	○	○	○	○
By using the system my gait will appear more normal to the general public	○	○	○	○	○

3.4. Facilitating conditions

	Strongly disagree	Disagree	Neutral	Agree	Strongly agree
There will be enough information explaining how to use the system	○	○	○	○	○
Using the system is compatible with my activities of daily life	○	○	○	○	○
I expect the system to adapt to any walking scenario	○	○	○	○	○

4. Importance of gait performance (open-ended)

4.1. For which daily-life activities would you like to improve gait performance?

5. Current problems of support devices to be used in daily-life activities

5.1. What changes to the current exoskeletons are needed to improve walking in daily-life situations?

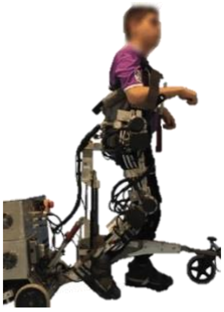

5.2. What changes to the current AFOs are needed to improve walking in daily-life situations?

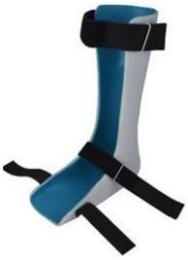

Supplement: Supplementary file 2 — Additional file 2. Final version of the English survey for end-users with CP and families (GU). [file 12984_2023_1162_MOESM2_ESM.pdf]
